# Divergent Soybean Calmodulins Respond Similarly to Calcium Transients: Insight into Differential Target Regulation

**DOI:** 10.3389/fpls.2017.00208

**Published:** 2017-02-15

**Authors:** Shane D. Walton, Harshini Chakravarthy, Vikram Shettigar, Andrew J. O’Neil, Jalal K. Siddiqui, Benjamin R. Jones, Svetlana B. Tikunova, Jonathan P. Davis

**Affiliations:** Department of Physiology and Cell Biology, The Ohio State UniversityColumbus, OH, USA

**Keywords:** calmodulin (CaM), calcium, magnesium, soybean, differential regulation

## Abstract

Plants commonly respond to stressors by modulating the expression of a large family of calcium binding proteins including isoforms of the ubiquitous signaling protein calmodulin (CaM). The various plant CaM isoforms are thought to differentially regulate the activity of specific target proteins to modulate cellular stress responses. The mechanism(s) behind differential target activation by the plant CaMs is unknown. In this study, we used steady-state and stopped-flow fluorescence spectroscopy to investigate the strategy by which two soybean CaMs (sCaM1 and sCaM4) have evolved to differentially regulate NAD kinase (NADK), which is activated by sCaM1 but inhibited by sCaM4. Although the isolated proteins have different cation binding properties, in the presence of Mg^2+^ and the CaM binding domains from proteins that are differentially regulated, the two plant CaMs respond nearly identically to rapid and slow Ca^2+^ transients. Our data suggest that the plant CaMs have evolved to bind certain targets with comparable affinities, respond similarly to a particular Ca^2+^ signature, but achieve different structural states, only one of which can activate the enzyme. Understanding the basis for differential enzyme regulation by the plant CaMs is the first step to engineering a vertebrate CaM that will selectively alter the CaM signaling network.

## Introduction

Ca^2+^ is a universal second messenger that influences nearly every function eukaryotic cells perform (Petersen et al., 2005; Clapham, 2007; Davis et al., 2016). The intensity and frequency of a particular Ca^2+^ dependent function is in part encoded by the amplitude, duration and frequency of the Ca^2+^ signal (Berridge and Galione, 1988). Another major influence on the response of the cell to Ca^2+^ is the Ca^2+^ sensing ability of the protein machinery that performs the function (Dupont et al., 2003; Dodd et al., 2010; Mehta et al., 2014; Shettigar et al., 2016). Considering the wide assortment of Ca^2+^ signals and the broad spectrum of Ca^2+^ dependent functions that can occur within a single cell, let alone different cells, it is not surprising there is a plethora of calcium binding proteins, each tuned to respond to particular Ca^2+^ patterns (Dodd et al., 2010; Batistič and Kudla, 2012; Davis et al., 2016).

In particular, all eukaryotic cells express the quintessential Ca^2+^ binding protein calmodulin (CaM) (Klee et al., 1980; Linse et al., 1991; Means et al., 1991). This small acidic protein binds and regulates a vast assortment of proteins that are also involved in nearly every cellular function (Cheung, 1980; Davis et al., 2016). Humans have three genes that encode an identical protein copy of CaM that is nearly invariant across all vertebrates (Toutenhoofd and Strehler, 2000; Friedberg and Rhoads, 2001). CaM’s Ca^2+^-binding properties are tuned to a wide spectrum of Ca^2+^ signals depending on its protein binding partner (Linse and Forsén, 1995; Maximciuc et al., 2006; Hoffman et al., 2014). In the human, dysregulation of several CaM-dependent enzymes and ion channels are at the root of several debilitating and devastating diseases such as arrhythmias, Alzheimer’s and hypertension (Xu et al., 2010; He et al., 2011; Nyegaard et al., 2012; O’Day et al., 2015). We are attempting to rewire the CaM signaling network by designing CaMs to correct for the dysregulated protein causing disease (Walton et al., 2016). Clues on how to smartly formulate targeted therapeutic CaMs will come from how the plant has evolved to handle stress (Walton et al., 2016).

Unlike vertebrates, plant species express several unique isoforms of CaM that can vary in sequence identity by as little as a single amino acid to approximately one quarter of the protein (McCormack and Braam, 2003; McCormack et al., 2005). Of the hundreds of unique CaM isoforms found *in plantae*, the vast majority differ by only a few amino acids. Some of these CaM isoforms are only expressed during times of stress (Al-Quraan, 2008; Park et al., 2009). One of the best characterized stress-induced CaM is found in the soybean, sCaM4. Compared to the “housekeeping” soybean CaM, sCaM1, sCaM4 binds nearly all the same target proteins, competitively inhibiting a select group of enzymes and ion channels that sCaM1 activates (Lee et al., 1995, 2000; Cho et al., 1998; Kondo et al., 1999).

Calmodulin is a versatile protein that can adopt numerous structural states depending on the intracellular concentration of Ca^2+^ as well as the various proteins and complexes within a cell for CaM to bind and interact (Tidow and Nissen, 2013; Kursula, 2014; Villarroel et al., 2014). CaM contains two globular domains connected by a flexible tether. In the absence of Ca^2+^, each globular domain is compact and relatively inert (although there are a growing number of proteins that can bind a Ca^2+^-free domain of CaM) (Putkey et al., 2003; Trybus, 2008). However, intracellular free Ca^2+^ in a resting or unstimulated cell is generally high enough such that at least one of the domains of CaM remains bound, but does not activate, target proteins (Van Lierop et al., 2002; Davis et al., 2016). Thus, the majority of CaM is pre-bound to its numerous targets and there is very little free CaM in most cells (Persechini et al., 1996; Wu and Bers, 2007). In order for CaM to activate or regulate its targets, Ca^2+^ levels must rise further to activate both domains of CaM, leading to a new active state structure. Somehow plants have evolved CaMs to be competitive antagonists of specific targets/enzymes (Lee et al., 1995, 2000; Cho et al., 1998; Kondo et al., 1999).

It has recently been suggested that the two soybean CaMs’ different Ca^2+^ and Mg^2+^ binding affinities are the reason for their differential target activation (Gifford et al., 2013). In this manuscript, we demonstrate that in the presence of a physiological concentration of Mg^2+^ and CaM binding domains from proteins that are differentially regulated, the two plant CaMs respond nearly identically to rapid and slow Ca^2+^ transients. Our data suggest that when bound to a target protein, the plant CaMs have evolved to respond similarly to a particular Ca^2+^ signature, but achieve different structural states, only one of which can activate the enzyme.

## Materials and Methods

### Materials

Phenyl-sepharose CL-4B, EDTA and EGTA were purchased from Sigma Chemical Co. (St. Louis, MO, USA). Quin-2 was purchased from Calbiochem (La Jolla, CA, USA). Bis-ANS and IAANS were purchased from Invitrogen (Carlsbad, CA, USA). All other chemicals were of analytical grade.

Ohio Peptide LLC (Powell, OH) synthesized the CaM binding domain of *Arabidopsis thaliana* NADK2 (IYVHSKEGVWRTSAMVSRWK) (Turner et al., 2004). CelTek Peptides (Franklin, TN, USA) synthesized the CaM binding domain of human myosin light chain kinase (MLCK) (ARRKWQKTGNAVRAIGRLSS) (Garcia et al., 1997).

We used three intact CaM isoforms: vertebrate CaM (CaM (although we used a bacterially codon optimized rat CaM gene, the protein sequence is identical in all vertebrates); soybean CaM isoform 1 (sCaM1); soybean CaM isoform 4 (sCaM4). The DNA constructs for CaM^T5C^, CaM^F19W^, sCaM1^F19W^, and sCaM4^F19W^ were generated from the respective wild type (WT) constructs in the pET17b vector (Studier and Moffatt, 1986; Black et al., 2000). Primer-selected site-directed mutagenesis was performed using the QuikChange Lightning Multi-site kit from Agilent (Santa Clara, CA, USA) following the manufacturer’s PCR protocol. All primers were synthesized by Integrated DNA Technologies (Coralville, IA, USA). Following PCR, the identity of each mutant was confirmed by DNA sequence analysis (GeneWiz). All CaMs were expressed in BL21 DE3 *Escherichia coli* and purified via phenyl-sepharose chromatography as previously described (Black et al., 2000).

We used several different fluorescent techniques to follow Ca^2+^ and Mg^2+^ binding and exchange with the different CaM constructs. Intrinsic tyrosine (Tyr) fluorescence can be used to follow the structural changes that occur within the C-domain of the WT CaMs (Black et al., 2000; Tikunova et al., 2001). We utilized the F19W mutation to exclusively follow the N-domain structural change via tryptophan (Trp) fluorescence, which occurs upon cation binding (Black et al., 2000; Tikunova et al., 2001). In order to observe the *N*- and C-domain pocket opening upon Ca^2+^-binding we utilized the fluorescent hydrophobic dyes BIS-ANS and 2,6-ANS (Fink, 1995). In order to directly follow Ca^2+^ dissociation from the CaM constructs we utilized the fluorescent Ca^2+^ chelator quin-2 (Davis et al., 2002). In order to follow WT CaM binding to their target peptides, we utilized the intrinsic Trp fluorescence of the peptides. We also utilized extrinsic fluorescent labeling of the native Cys residue in the two plant CaMs and engineered a single Cys mutant (T5C) in the vertebrate CaM and labeled the constructs with the environmentally sensitive fluorophore IAANS. IAANS fluorescence was sensitive to both N- and C-domain Ca^2+^-binding to the CaM constructs in the presence of the CaM target peptides.

### Steady-State Measurements

The steady-state fluorescence measurements were performed using a Perkin-Elmer LS55 spectrofluorimeter at 20°C. The titration buffer consisted of 200 mM MOPS, 150 mM KCl, 2 mM EGTA (pH 7.0) (Tikunova et al., 2002, 2010; Tikunova and Davis, 2004). In order to follow the Ca^2+^-dependent change in the N-terminal domain of CaM, Trp fluorescence was excited at 295 nm and monitored at 350 nm. In order to follow the Ca^2+^-dependent change in the C-terminal domain of CaM, Tyr fluorescence was excited at 275 nm and monitored at 305 nm. The free Ca^2+^ concentration was calculated using the EGCA02 program by Robertson and Potter (1984). The free Mg^2+^ concentration was calculated using the MaxChelator program (Bers et al., 2010). Each reported *K*_d_ represents an average of at least three successive titrations ± standard error and fit with the Hill equation as previously described (George et al., 1996).

To determine the affinity of each CaM for Ca^2+^ or Mg^2+^, fluorescence emission intensity was recorded as microliter amounts of CaCl_2_ or MgCl_2_ were added to 2 mL of each CaM (1 μM) in titration buffer with constant stirring (Tikunova et al., 2002; Zhang et al., 2011). The Ca^2+^ titrations in the presence of Mg^2+^ were performed as described above with the addition of 3 mM Mg^2+^ to the titration buffer. To determine the affinity of each CaM isoform for NADK, each CaM was titrated into 2 mL of titration buffer containing 1 μM NADK and 100 μM Ca^2+^ (pCa = 4) (Johnson et al., 1996). The Ca^2+^ titrations in the presence of NADK or MLCK peptide were performed with 4 μM peptide in the 1 μM CaM solution.

### Kinetic Measurements

The kinetic data were collected using an Applied Photophysics Ltd. (Leatherhead, UK) model SX.18 MV stopped-flow instrument with a dead time of 1.4 ms at 20°C. The samples were excited using a 150W xenon arc source (Tikunova et al., 2002; Davis et al., 2004, 2007). The stopped-flow buffer for all experiments consisted of 10 mM MOPS, 150 mM KCl (pH 7.0). Tyr fluorescence was excited at 275 nm and emission was monitored using a UV-transmitting black glass (UG1) filter from Oriel (Stratford, CT) (VanScyoc et al., 2002). Trp fluorescence was excited at 295 nm and also monitored using a UG1 filter (Davis et al., 2004). Bis-ANS fluorescence was excited at 390 nm and emission monitored using a 500 nm long pass interference filter from Newport (Irvine, CA, USA) (Hawe et al., 2008). 2,6-ANS or IAANS fluorescence were excited at 320 or 330 nm, respectively, and emission for both probes was monitored using a 420–470 nm band pass interference filter from Oriel (Stratford, CT, USA) (Tikunova et al., 2010). Quin-2 fluorescence was excited at 330 nm and emission was monitored using a 510 nm broad band pass interference filter from Oriel (Stratford, CT, USA) (Tikunova et al., 2002).

In order to measure rates of Ca^2+^ dissociation via structural change, stopped-flow buffer with 200 μM Ca^2+^ and either 1 μM CaM^F19W^ (Trp) or 3 μM CaM (Tyr) was rapidly mixed with 10 mM EGTA in stopped-flow buffer. To measure the Ca^2+^-dependent rate of hydrophobic pocket closure, stopped-flow buffer with 200 μM Ca^2+^, 1 μM CaM and 0.5 μM bis-ANS was rapidly mixed with 10 mM EGTA in stopped-flow buffer. For the actual Ca^2+^ dissociation rate measurements, stopped-flow buffer with 30 μM Ca^2+^ and 6 μM CaM was rapidly mixed with 150 μM quin-2 in stopped-flow buffer. To directly determine the rate of Ca^2+^ dissociation in the presence of peptide, stopped-flow buffer with 30 μM Ca^2+^, 6 μM CaM and 18 μM peptide was rapidly mixed with 150 μM quin-2 in stopped-flow buffer. To determine the rate of Ca^2+^ dissociation via structural change in the presence of peptide, stopped-flow buffer with 1 μM IAANS-labeled CaM, 200 μM Ca^2+^, and 3 μM NADK or MLCK peptide was rapidly mixed with 10 mM EGTA in stopped-flow buffer. Labeling of CaM^T5C^, WT sCaM1, and WT sCaM4 was performed as previously described, taking advantage of the native C26 in the soybean CaMs (Tikunova et al., 2010). To determine rate of Ca^2+^ dissociation in the presence of peptide via the intrinsic Trp in each peptide, 1 μM CaM, 200 μM Ca^2+^, and 5 μM NADK or MLCK peptide in stopped-flow buffer was rapidly mixed with 10 mM EGTA in stopped-flow buffer.

For the Mg^2+^ dissociation experiments, stopped-flow buffer with 3 mM Mg^2+^, 1 μM CaM^F19W^ and 15 μM EGTA (to chelate contaminating Ca^2+^) was rapidly mixed with 30 mM EDTA in stopped-flow buffer. The kinetics of Ca^2+^/Mg^2+^ competition were determined using F19W fluorescence. Stopped-flow buffer with 1 μM CaM^F19W^, 3 mM Mg^2+^, and 15 μM EGTA was rapidly mixed with solution containing 2 mM Ca^2+^ in stopped-flow buffer. The competition experiments with 2,6-ANS rapidly mixed solution containing stopped-flow buffer, 2 μM CaM, 3 mM Mg^2+^, 15 μM EGTA and 1 μM 2,6-ANS with solution containing 2 mM Ca^2+^ in stopped-flow buffer.

Rapid Ca^2+^ transients were generated by mixing a solution containing stopped-flow buffer, 2 μM of each sCaM^F19W^ mutant and 2 mM EGTA with solution containing stopped-flow buffer, 0, 100 μM, 250 μM or 2 mM Ca^2+^ in both the presence and absence of 3 mM Mg^2+^. We followed the binding and subsequent dissociation of Ca^2+^ at the N-domain of CaM or its isoforms using fluorescence from the engineered Trp residue. The visible percent occupancy was calculated using 0 mM Ca^2+^ as the baseline and 2 mM Ca^2+^ as the maximum.

Slow Ca^2+^ transients were generated by mixing solution containing stopped-flow buffer, 3 μM of each sCaM isoform, 9 μM NADK, and 500 μM EDTA with solution containing stopped-flow buffer, 0, 10, 30, 60, 120, and 250 μM, or 2 mM Ca^2+^ in the presence of 1 mM Mg^2+^. As for the rapid transients, the visible percent occupancy was calculated using 0 mM Ca^2+^ as the baseline and 2 mM Ca^2+^ as the maximum. We followed the binding and subsequent dissociation of Ca^2+^ using Trp from the NADK peptide.

Bis-ANS fluorescence was used as described above to follow the structural change as Ca^2+^ dissociates from the N-domain of sCaM1 or sCaM4. The amplitudes from the Ca^2+^ dissociation without peptide were set at 100% and the amplitudes in the presence of increasing amounts of NADK (3, 6, and 15 μM) were then expressed as a percentage of this peptide-free amplitude.

All kinetic data were fit with a program (by P. J. King, Applied Photophysics Ltd.) that uses the non-linear Levenberg–Marquardt algorithm (Tikunova et al., 2010). Each *k*_off_ value represents an average of at least three separate experiments, each averaging at least five traces fit with either a single exponential equation (Trp, Tyr) or a double exponential equation (bis-ANS, quin-2) (Tikunova et al., 2002).

### Statistical Analysis

All values are expressed as mean ± SEM. Statistical significance (*p* < 0.05) was determined by one-way ANOVA followed by *post hoc* analysis with Tukey’s test using the statistical analysis software MiniTab16 (State College, PA, USA). Statistical significance (*p* < 0.05) for the rapid and slow transient occupancy experiments was determined by two-way ANOVA.

## Results

### Ca^2+^ Binding to the Isolated Plant CaMs

**FIGURE 1:**
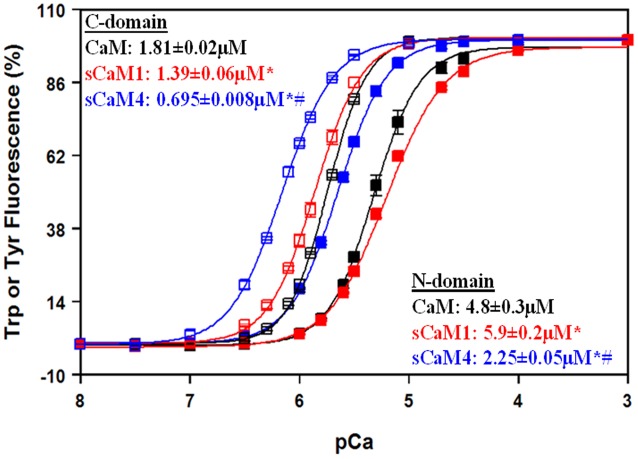
**Domain-specific Ca^2+^ sensitivities for isolated CaMs.** The Ca^2+^-dependent increase in Trp (N-domain, closed squares) or Tyr (C-domain, open squares) fluorescence is shown as a function of -log[Ca^2+^] for CaM (black), sCaM1 (red) and sCaM4 (blue). Increasing amounts of Ca^2+^ were added to a 2 mL solution containing 1 μM of each protein (F19W for Trp, WT for Tyr) in 200 mM MOPS, 150 mM KCl, 2 mM EGTA, pH 7.0 at 20°C. Significant difference (*p* < 0.05) of sCaM1 and sCaM4 from CaM is denoted by (^∗^) and significant difference of sCaM4 from sCaM1 is denoted by (#). The traces were fit and the free [Ca^2+^] calculated as described in section “Materials and Methods”.

**Figure 1** shows the steady-state Ca^2+^ binding curves from the C-domain of WT CaM isoforms (open squares; Tyr fluorescence) and from the N-domain of F19W CaM (Black et al., 2000; Tikunova et al., 2001) isoforms (solid squares; Trp fluorescence). Consistent with recent NMR data (Gifford et al., 2013), the C-domains for each soybean CaM had approximately fourfold higher Ca^2+^ affinity than the respective N-domains, which was also true for vertebrate CaM. In addition, both the N- and C-domains of sCaM4 had approximately two–threefold higher Ca^2+^ affinity than that of sCaM1 (Gifford et al., 2013). Considering steady-state behavior can be modulated by tuning the rate of Ca^2+^ exchange (Martin et al., 1992; Tikunova et al., 2010), it is important to understand the kinetic parameters of the CaM isoforms. Furthermore in a cell, Ca^2+^ levels are rarely in steady-state and rise and fall transiently.

### Ca^2+^ Dissociation from the Isolated Plant CaMs

**FIGURE 2:**
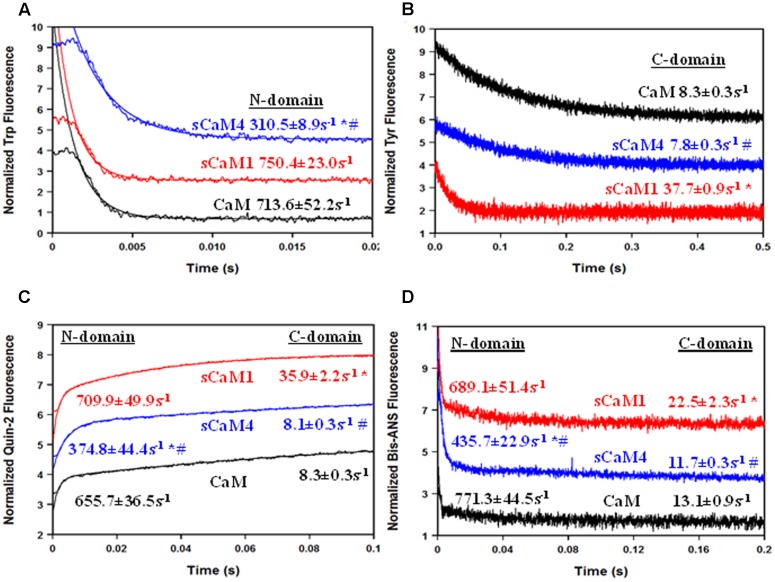
**Ca^2+^ Dissociation from CaM, sCaM1, and sCaM4. (A)** Time course of the change in Trp fluorescence as Ca^2+^ is removed from CaM (black), sCaM1 (blue), and sCaM4 (black) by EGTA. Each F19W protein (1 μM) with 200 μM Ca^2+^ in stopped-flow buffer (10 mM MOPS, 150 mM KCl, pH 7.0) was rapidly mixed with an equal volume of 10 mM EGTA in stopped-flow buffer at 20°C. **(B)** Time course of the change in Tyr fluorescence as Ca^2+^ is removed from each CaM by EGTA. Each WT protein (3 μM) with 200 μM Ca^2+^ in stopped-flow buffer was rapidly mixed with an equal volume of 10 mM EGTA in stopped-flow buffer at 20°C. **(C)** Time course of the change in quin-2 fluorescence as Ca^2+^ is removed from each CaM by quin-2. Each F19W protein (6 μM) with 30 μM Ca^2+^ in stopped-flow buffer was rapidly mixed with an equal volume of 150 μM quin-2 in stopped-flow buffer at 20°C. **(D)** Time course of the change in bis-ANS fluorescence as Ca^2+^ is removed from each CaM by EGTA. Each WT protein (1 μM) with 200 μM Ca^2+^ and 0.5 μM bis-ANS in stopped-flow buffer was rapidly mixed with an equal volume of 10 mM EGTA in stopped-flow buffer at 20°C. Significant difference (*p* < 0.05) of sCaM1 and sCaM4 from CaM is denoted by (^∗^) and significant difference of sCaM4 from sCaM1 is denoted by (#). All traces in this figure were normalized and displaced vertically for clarity, and were fit as described in section “Materials and Methods.”

Steady-state Ca^2+^ binding is defined by how quickly the domain can associate and dissociate Ca^2+^. **Figure 2A** shows the Ca^2+^ dissociation rates from the N-domain of the F19W CaM isoforms using Trp fluorescence. Consistent with the differences in Ca^2+^ affinity, the N-domain of sCaM4 had a ∼2.5-fold slower rate of Ca^2+^ dissociation compared to that of sCaM1 (**Table 1**). **Figure 2B** shows the Ca^2+^ dissociation rates from the C-domain of the WT CaM isoforms using Tyr fluorescence. Ca^2+^ dissociated from the C-domain of sCaM4 approximately fivefold more slowly than from sCaM1. Thus, Ca^2+^ dissociates from the N-domain faster than from the C-domain for both plant CaM isoforms, again in agreement with vertebrate CaM behavior and consistent with the higher Ca^2+^ affinity of the C-domain. **Figure 2C** shows the actual rate of Ca^2+^ dissociation from both domains of each F19W CaM using the high-affinity fluorescent Ca^2+^ chelator quin-2 (Martin et al., 1985; Tikunova et al., 2002). For all the CaMs, the actual Ca^2+^ dissociation rates were nearly identical to those reported by the local structural change sensed by the intrinsic fluorophores at each individual domain. **Figure 2D** shows the rate of hydrophobic pocket closure in the CaMs as Ca^2+^ dissociates, using the hydrophobic fluorescent dye bis-ANS. For all the CaMs, the rate of hydrophobic pocket closure is comparable to the rate of structural change reported by the intrinsic fluorophores at each domain, which occurs at the actual Ca^2+^ dissociation rate. Although it would appear from the steady-state data (**Figure 1**) that sCaM1 behaves more similarly to CaM, kinetically this appears to be true only for the N-terminal domain, whereas kinetically the C-terminal domain of sCaM4 appears more similar to CaM (**Figure 2**; **Table 1**). Overall, sCaM4 had a higher Ca^2+^ affinity and slower Ca^2+^ dissociation rates than sCaM1, suggesting that differences in Ca^2+^ binding might be one strategy the plants have used to tune CaM function (Gifford et al., 2013).

**Table 1:** Ca^2+^ Dissociation rates for vertebrate and plant CaM isoforms.

Protein	N-Domain			C-domain		
	TrpCa^2+^ *k*_off_	Quin-2Ca^2+^ *k*_off_	Bis-ANSCa^2+^ *k*_off_	TyrCa^2+^ *k*_off_	Quin-2Ca^2+^ *k*_off_	Bis-ANSCa^2+^ *k*_off_
CaM	713.6 ± 52.2s^-1^	655.7 ± 36.5s^-1^	771.3 ± 44.5s^-1^	8.3 ± 0.3s^-1^	8.3 ± 0.3s^-1^	13.1 ± 0.9s^-1^
sCaM1	750.4 ± 23.0s^-1^	709.9 ± 49.9s^-1^	689.1 ± 51.4s^-1^	37.7 ± 0.9s^-1∗^	35.9 ± 2.2s^-1∗^	22.5 ± 2.3s^-1∗^
sCaM4	310.5 ± 8.9s^-1∗^#	374.8 ± 44.4s^-1∗^#	435.7 ± 22.9s^-1∗^#	7.8 ± 0.3s^-1^ #	8.1 ± 0.3s^-1^#	11.7 ± 0.3s^-1^#

	**N-domain with NADK**			**C-domain with NADK**		
	**TrpCa**^2+^ ***k*_off_**	**Quin-2Ca**^2+^ ***k*_off_**	**IAANSCa**^2+^ ***k*_off_**	**Trp Ca**^2+^ ***k*_off_**	**Quin-2 Ca**^2+^ ***k*_off_**	**IAANSCa**^2+^ ***k*_off_**
CaM	40.7 ± 6.8s^-1^	27.0 ± 0.7s^-1^	29.5 ± 0.6s^-1^	5.2 ± 0.6s^-1^	3.9 ± 0.1s^-1^	2.66 ± 0.06s^-1^
sCaM1	43.6 ± 1.2s^-1^	22.1 ± 0.9s^-1^	85.2 ± 2.6s^-1∗^	8.7 ± 0.8s^-1^	3.7 ± 0.5s^-1^	7.2 ± 0.6s^-1∗^
sCaM4	80.5 ± 4.5s^-1^#	62.5 ± 2.7s^-1∗^#	48.8 ± 3.8s^-1∗^#	7.7 ± 0.8s^-1^	5.8 ± 0.2s^-1∗^#	9.4 ± 0.9s^-1∗^#

	**N-domain with MLCK**			**C-domain with MLCK**		
	**TrpCa**^2+^ ***k*_off_**	**Quin-2Ca**^2+^ ***k*_off_**	**IAANSCa**^2+^ ***k*_off_**	**TrpCa**^2+^ ***k*_off_**	**Quin-2Ca**^2+^ ***k*_off_**	**IAANSCa**^2+^ ***k*_off_**
CaM	N/A	6.1 ± 0.1s^-1^	8.40 ± 0.05s^-1^	0.67 ± 0.01s^-1^	0.35 ± 0.01s^-1^	0.683 ± 0.005s^-1^
sCaM1	N/A	3.2 ± 0.2s^-1∗^	6.17 ± 0.07s^-1∗^	0.8 ± 0.1s^-1^	0.40 ± 0.03s^-1^	1.02 ± 0.04s^-1∗^
sCaM4	N/A	3.1 ± 0.1s^-1∗^	4.89 ± 0.07s^-1∗^#	0.94 ± 0.07s^-1^	0.65 ± 0.03s^-1∗^#	1.33 ± 0.07s^-1∗^#

*Kinetic data are summarized. Significant difference (*p* < 0.05) of sCaM1 and sCaM4 from CaM is denoted by (^∗^) and significant difference of sCaM4 from sCaM1 is denoted by (##)*.

### Mg^2+^ Binding to the Isolated Plant CaMs

**FIGURE 3:**
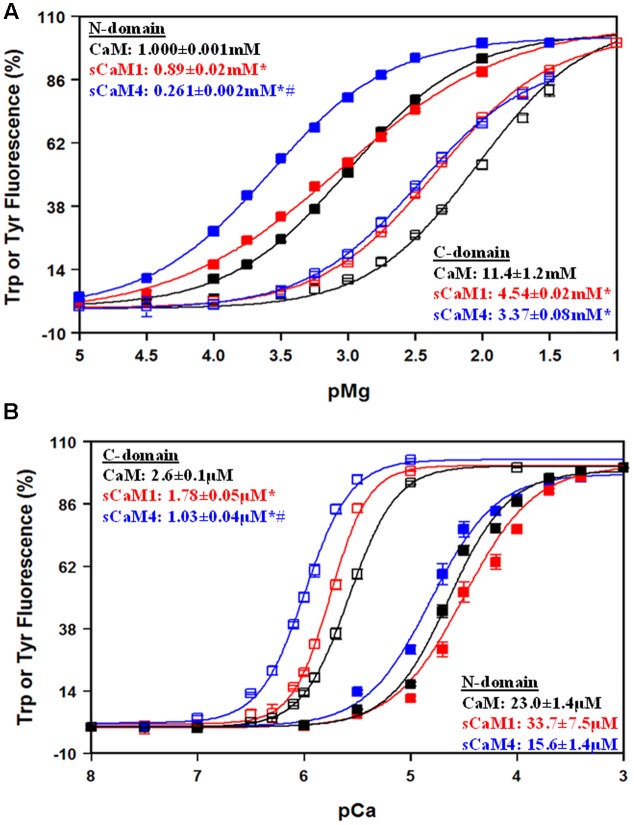
**Mg^2+^ Sensitivity for CaM, sCaM1, and sCaM4. (A)** Mg^2+^-dependent change in Trp (N-domain, closed squares) or Tyr (C-domain, open squares) fluorescence as a function of -log[Mg^2+^] for CaM (black), sCaM1 (red), or sCaM4 (blue). Increasing amounts of Mg^2+^ were added to 2 mL containing 1 μM of each protein (F19W for Trp, WT for Tyr) in 200 mM MOPS, 150 mM KCl, 2 mM EGTA, pH 7.0 at 20°C. The free [Mg^2+^] was calculated as described in section “Materials and Methods.” **(B)** Ca^2+^-dependent change in Trp (N-domain, closed squares) or Tyr (C-domain, open squares) fluorescence as a function of -log[Ca^2+^]. Increasing amounts of Ca^2+^ were added to 2 mL containing 1 μM of each protein (F19W for Trp, WT for Tyr) with 3 mM Mg^2+^ in the same buffer as **(A)** at 20°C. Significant difference (*p* < 0.05) of sCaM1 and sCaM4 from CaM is denoted by (^∗^) and significant difference of sCaM4 from sCaM1 is denoted by (#). The free [Ca^2+^] was calculated as described in section “Materials and Methods.” All traces were fit and affinities calculated as described in section “Materials and Methods.”

In addition to Ca^2+^, CaM is known to bind Mg^2+^ competitively, competing with up to 1 mM free Mg^2+^ in the cell (Ohki et al., 1997; Tikunova et al., 2001; Waters, 2011; Gifford et al., 2013). In agreement with previous findings, our data show the N-domain of all the CaMs had a physiologically relevant Mg^2+^ affinity, whereas the C-domain Mg^2+^ affinity falls well outside the physiological range (**Figure 3A**). The Mg^2+^ affinity of the N-domain of sCaM4^F19W^ was approximately threefold higher compared to sCaM1^F19W^, but not significantly different at the C-domain. Based on these data, Mg^2+^should drastically decrease the apparent Ca^2+^ sensitivity of the N-domain while only slightly affecting the C-domain. **Figure 3B** shows the effect of 3 mM Mg^2+^ on Ca^2+^ binding to the vertebrate and soybean CaMs. As expected, the apparent Ca^2+^ sensitivity of the N-domain of all the CaMs decreased approximately sixfold in the presence of Mg^2+^. On the other hand, the apparent C-domain Ca^2+^ sensitivity for all the CaMs only marginally decreased by ∼1.5-fold. Thus, in a cell the high level of Mg^2+^ would be expected to exert a greater effect of competing for Ca^2+^ binding to the N-domain of CaM, with little effect on the C-terminal domain Ca^2+^ binding.

### Mg^2+^ Dissociation from Isolated CaMs

**FIGURE 4:**
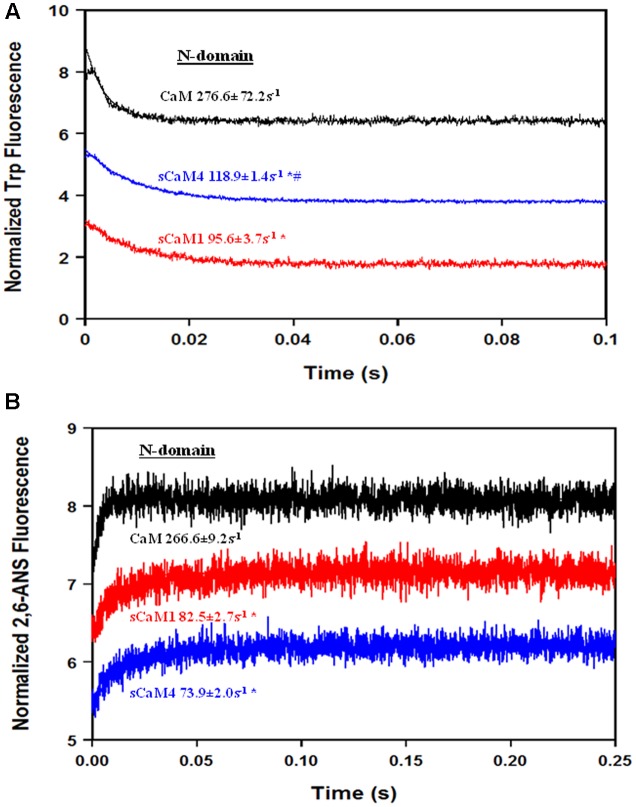
**Mg^2+^ dissociation from CaM, sCaM1, and sCaM4. (A)** Time course of the change in Trp fluorescence as Mg^2+^ is removed from CaM (black), sCaM1 (red), and sCaM4 (blue) by EDTA. 1 μM of each F19W protein with 15 μM EGTA and 3 mM Mg^2+^ in stopped-flow buffer (10 mM MOPS, 150 mM KCl, pH 7.0) was rapidly mixed with an equal volume of 30 mM EDTA in stopped-flow buffer at 20°C. **(B)** Time course of the change in 2,6-ANS fluorescence as Mg^2+^ is removed from CaM, sCaM1, and sCaM4 by Ca^2+^. 2 μM of each WT protein with 1 μM 2,6-ANS, 15 μM EGTA and 3 mM Mg^2+^ in stopped-flow buffer was rapidly mixed with an equal volume of 2 mM Ca^2+^ in stopped-flow buffer at 20°C. Significant difference (*p* < 0.05) of sCaM1 from CaM is denoted by (^∗^) and significant difference from of sCaM4 from sCaM1 is denoted by (#). All traces in this figure were normalized and displaced vertically for clarity, and were fit as described in section “Materials and Methods.”

In order for Ca^2+^ to bind to the N-domain of CaM in the presence of Mg^2+^, Mg^2+^ must first dissociate (Tikunova et al., 2001), adding another layer of regulation for Ca^2+^ sensing (Davis et al., 2016). **Figure 4A** shows the rate of Mg^2+^ dissociation from the N-domain of the F19W CaM isoforms using Trp fluorescence. Despite having distinct N-terminal Mg^2+^ affinities, the soybean CaMs had nearly identical Mg^2+^ dissociation rates. Based on these data, Mg^2+^ should drastically slow the rate of N-domain pocket opening in response to a rise in Ca^2+^. **Figure 4B** shows the rate of N-domain pocket opening for the CaMs using 2,6-ANS. As expected, the rate of N-domain pocket opening is now limited by the rate of Mg^2+^ dissociation. These data suggest that Mg^2+^ would drastically affect the ability of the N-domain to respond to a rapid Ca^2+^ transient.

### Exposure of the Isolated Plant CaMs to Rapid Ca^2+^ Transients

**FIGURE 5:**
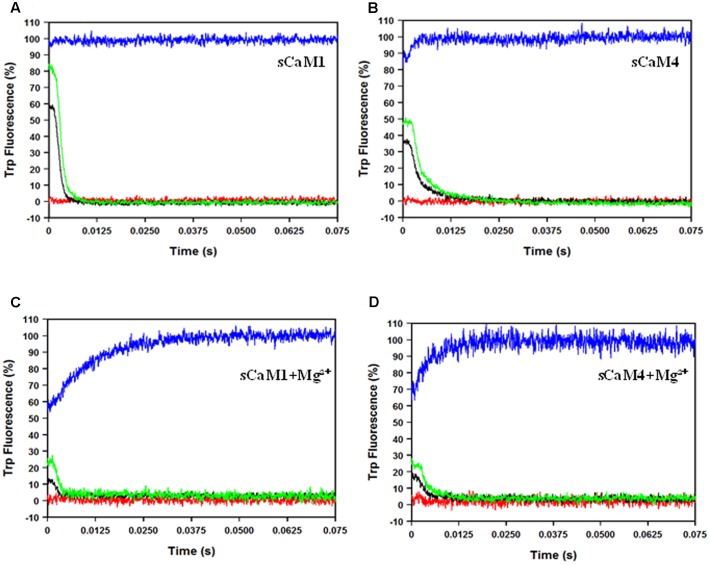
**sCaM1 and sCaM4 N-terminal response to rapid Ca^2+^ transients. (A)** Time course of the change in Trp fluorescence as sCaM1 is transiently occupied by Ca^2+^. 2 μM sCaM1^F19W^ with 2 mM EGTA in stopped-flow buffer (10 mM MOPS, 150 mM KCl, pH 7.0) was rapidly mixed with an equal volume of increasing Ca^2+^ (0 μM (red), 100 μM (black), 250 μM (green), 2.2 mM (blue) in stopped-flow buffer at 20°C. **(B)** Time course of the change in Trp fluorescence as sCaM4 is transiently occupied by Ca^2+^. 2μM sCaM4^F19W^ with 2 mM EGTA in stopped-flow buffer was rapidly mixed with an equal volume of increasing Ca^2+^ (0 μM, 100 μM, 250 μM, 2.2 mM) in stopped-flow buffer at 20°C. **(C)** Time course of the change in Trp fluorescence as sCaM1 is transiently occupied by Ca^2+^ in the presence of Mg^2+^. 2 μM sCaM1^F19W^ with 3 mM Mg^2+^ and 2 mM EGTA in stopped-flow buffer was rapidly mixed with an equal volume of increasing Ca^2+^ (0 μM, 100 μM, 250 μM, 2.2 mM) in stopped-flow buffer, plus 3 mM Mg^2+^ at 20°C. **(D)** Time course of the change in Trp fluorescence as sCaM4 is transiently occupied by Ca^2+^ in the presence of Mg^2+^. 2 μM sCaM4^F19W^ with 3 mM Mg^2+^ and 2 mM EGTA in stopped-flow buffer was rapidly mixed with an equal volume of increasing Ca^2+^ (0 μM, 100 μM, 250 μM, 2.2 mM) in stopped-flow buffer plus 3 mM Mg^2+^ at 20°C. Each trace is an average of at least three separate experiments, each averaging at least five traces, and the visible occupancy for all traces was determined as described in section “Materials and Methods.”

**Figures 5A,B** show the response of the N-domain of the plant CaMs to rapid (approximately 0.4 ms half-life) artificial Ca^2+^ transients (Davis et al., 1999). The percentage of sCaM4 able to bind Ca^2+^ during the rapid transients was significantly lower than that of sCaM1 (**Table 2**). **Figures 5C,D** show the response of the N-domain of the plant CaMs to rapid artificial transients in the presence of Mg^2+^. Consistent with their similar N-domain Mg^2+^ dissociation rates, in the presence of Mg^2+^ the two plant CaMs responded similarly to the rapid artificial Ca^2+^ transients. Thus, the addition of Mg^2+^ appears to minimize the differences in the Ca^2+^ sensing ability between the plant CaMs. Thus, in a cell the rate of Ca^2+^-binding to the N-domain of CaM will be limited by Mg^2+^ dissociation.

**Table 2:** Summary of sCaM1 and sCaM4 Responses to Ca^2+^ Transients.

Protein	Rapid100 μM Ca^2+^	Rapid250 μM Ca^2+^	Rapid + Mg^2+^100 μM Ca^2+^	Rapid + Mg^2+^250 μM Ca^2+^	
sCaM1	58.9 ± 1.1%	82.9 ± 0.5%	13.7 ± 0.3%	22.3 ± 0.5%	
sCaM4	44.1 ± 0.6%#	57.5 ± 0.3%#	17.3 ± 0.7%	25.0 ± 0.9%	

	**Slow + Mg**^2+^ **+NADK10μM Ca**^2+^	**Slow + Mg**^2+^ **+NADK30 μM Ca**^2+^	**Slow + Mg**^2+^ **+NADK60 μM Ca**^2+^	**Slow + Mg**^2+^ **+NADK120 μM Ca**^2+^	**Slow + Mg**^2+^ **+NADK250 μM Ca**^2+^

sCaM1	9.2 ± 0.8%	42.4 ± 1.3%	70.2 ± 1.2%	86.4 ± 0.9%	80.9 ± 2.4%
sCaM4	8.4 ± 0.5%	31.8 ± 1.6%	65.3 ± 2.2%	81.5 ± 2.8%	87.6 ± 2.2%

*The percentage of protein transiently occupied by Ca^*2*+^ for rapid and slow transients is summarized. Significant difference (*p* < 0.05) of sCaM4 from sCaM1 is indicated by ^#^*.

### Effects of Target Peptides on the Plant CaMs

**FIGURE 6:**
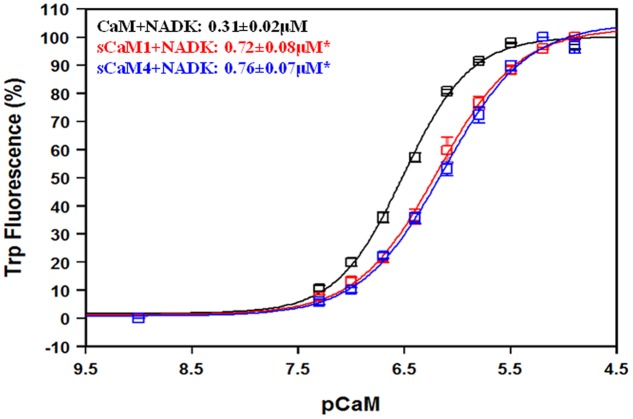
**NAD kinase affinity for CaM, sCaM1, and sCaM4.** The CaM-dependent change in fluorescence for the intrinsic Trp in NADK is shown as a function of -log[CaM] for CaM (black), sCaM1 (red), and sCaM4 (blue). Increasing amounts of each WT protein were added to 2 mL of buffer (200 mM MOPS, 150 mM KCl, 2 mM EGTA, pH 7.0) containing 1 μM NADK with 100 μM Ca^2+^ (pCa = 4) at 20°C. There was no fluorescence change for any of the CaMs in the absence of Ca^2+^ (data not shown). Significant difference (*p* < 0.05) of sCaM1 and sCaM4 from CaM is denoted by (^∗^). The free [Ca^2+^] was calculated as described in section “Materials and Methods.” All traces were fit and affinities calculated as described in section “Materials and Methods.”

Another critical element to understanding the differential regulation of these plant CaMs is their ability to bind targets. We chose to study this phenomenon using two peptides from enzymes known to be competitively inhibited by sCaM4, myosin light chain kinase (MLCK) and NAD kinase (NADK) (Lee et al., 2000). Currently NADK is the only plant enzyme known to be differentially regulated by the soybean CaMs (Lee et al., 1997; Turner et al., 2004). Furthermore, vertebrate CaM is a poor activator of NADK, but a potent activator of MLCK (Roberts et al., 1984). **Figure 6** shows the NADK peptide affinity for the Ca^2+^-saturated CaMs using the intrinsic Trp fluorescence of the peptide. Similar to MLCK (Van Lierop et al., 2002), both plant CaMs bound the NADK peptide with a nearly identical affinity, albeit with a twofold lower affinity than CaM. In agreement with previous work, NADK did not bind the CaMs in the absence of Ca^2+^ (data not shown) (Turner et al., 2004).

**FIGURE 7:**
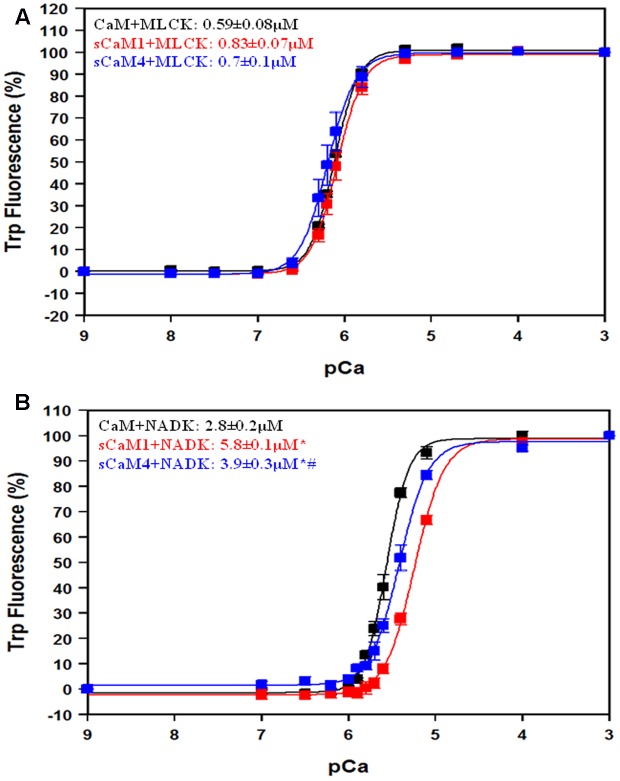
**Effect of MLCK, NADK peptides on Ca^2+^ sensitivity for CaM, sCaM1, and sCaM4. (A)** Ca^2+^-dependent change in fluorescence for the intrinsic Trp in MLCK as a function of -log[Ca^2+^] for CaM (black), sCaM1 (red), and sCaM4 (blue). Increasing amounts of Ca^2+^ were added to 2 mL buffer (200 mM MOPS, 150 mM KCl, 2 mM EGTA, pH 7.0) containing each WT protein (1 μM) with 4 μM MLCK at 20°C. **(B)** Ca^2+^-dependent change in fluorescence for the intrinsic Trp in NADK as a function of -log[Ca^2+^]. Increasing amounts of Ca^2+^ were added to 2 mL containing each WT protein (1 μM) with 4 μM NADK in the same buffer as **(A)** at 20°C. Significant difference (*p* < 0.05) of sCaM1 and sCaM4 from CaM is denoted by (^∗^) and significant difference of sCaM4 from sCaM1 is denoted by (#). The free [Ca^2+^] was calculated as described in section “Materials and Methods.” All traces were fit and affinities calculated as described in section “Materials and Methods.”

**Figures 7A,B** show the apparent Ca^2+^ sensitivity of the CaMs in the presence of MLCK and NADK peptides using the intrinsic Trp fluorescence of the peptides. In the presence of the MLCK peptide, there was no significant difference in apparent Ca^2+^ affinity between the CaMs. In the presence of the NADK peptide, the apparent Ca^2+^ affinity of sCaM4 was only ∼1.5-fold higher than that of sCaM1, but ∼1.5-fold lower than that of CaM. In general, the NADK peptide had a weaker affinity for the Ca^2+^-saturated CaMs and also had a substantially weaker effect at sensitizing the CaMs to Ca^2+^ compared to MLCK.

**FIGURE 8:**
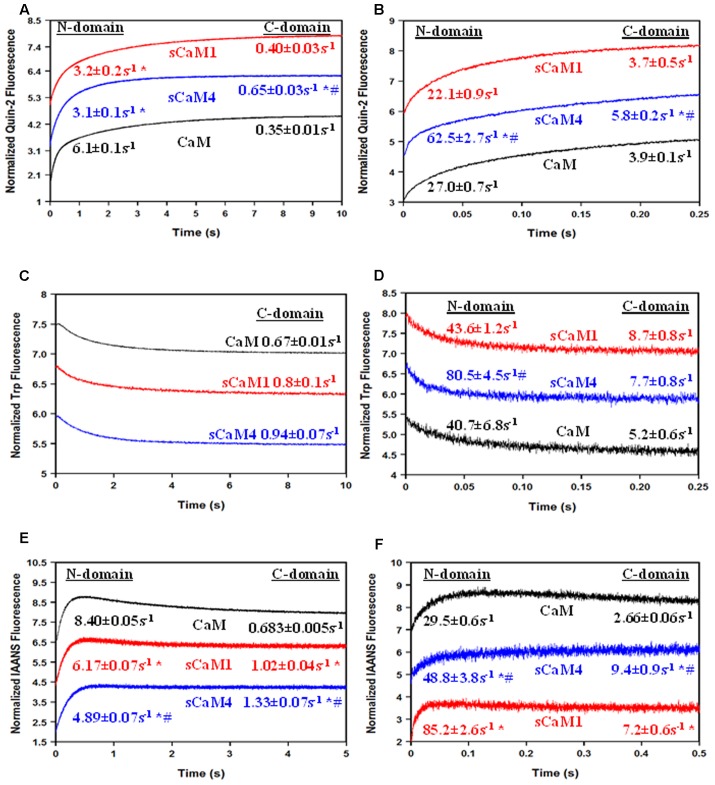
**Peptide effects on Ca^2+^ dissociation from CaM, sCaM1, and sCaM4. (A)** Time course of the change in quin-2 fluorescence as Ca^2+^ is removed from the WT CaM+MLCK complex by quin-2. Each protein (6 μM) with 30 μM Ca^2+^ and 18 μM MLCK in stopped-flow buffer (10 mM MOPS, 150 mM KCl, pH 7.0) was rapidly mixed with an equal volume of 150 μM quin-2 in stopped-flow buffer at 20°C. **(B)** Time course of the change in quin-2 fluorescence as Ca^2+^ is removed from the WT CaM+NADK complex by quin-2. Each protein (6 μM) with 30 μM Ca^2+^ and 18 μM NADK in stopped-flow buffer was rapidly mixed with an equal volume of 150 μM quin-2 in stopped-flow buffer at 20°C. **(C)** Time course of the change in peptide Trp fluorescence as Ca^2+^ is removed from the WT CaM+MLCK complex by EGTA. Each protein (1 μM) with 200 μM Ca^2+^ and 5 μM MLCK in stopped-flow buffer (10 mM MOPS, 150 mM KCl, pH 7.0) was rapidly mixed with an equal volume of 10 mM EGTA in stopped-flow buffer at 20°C. **(D)** Time course of the change in peptide Trp fluorescence as Ca^2+^ is removed from the WT CaM+NADK complex by EGTA. Each protein (1 μM) with 200 μM Ca^2+^ and 5 μM NADK in stopped-flow buffer (10 mM MOPS, 150 mM KCl, pH 7.0) was rapidly mixed with an equal volume of 10 mM EGTA in stopped-flow buffer at 20°C. **(E)** Time course of the change in IAANS fluorescence as Ca^2+^ is removed from the WT CaM+MLCK complex by EGTA. Each IAANS-labeled protein (1 μM) with 200 μM Ca^2+^ and 3 μM MLCK in stopped-flow buffer (10 mM MOPS, 150 mM KCl, pH 7.0) was rapidly mixed with an equal volume of 10 mM EGTA in stopped-flow buffer at 20°C. **(F)** Time course of the change in IAANS fluorescence as Ca^2+^ is removed from the WT CaM+NADK complex by EGTA. Each IAANS-labeled protein (1 μM) with 200 μM Ca^2+^ and 3 μM NADK in stopped-flow buffer (10 mM MOPS, 150 mM KCl, pH 7.0) was rapidly mixed with an equal volume of 10 mM EGTA in stopped-flow buffer at 20°C. Significant difference (*p* < 0.05) of sCaM1 and sCaM4 from CaM is denoted by (^∗^) and significant difference from of sCaM4 from sCaM1 is denoted by (#). All traces in this figure were normalized and displaced vertically for clarity, and were fit as described in section “Materials and Methods.”

**Figures 8A,B** show the actual Ca^2+^ dissociation rates from both domains of the CaMs bound to either MLCK or NADK peptides using quin-2 fluorescence. Surprisingly, unlike the MLCK peptide, NADK only marginally slowed the Ca^2+^ dissociation from the C-terminal domain of sCaM4 and vertebrate CaM (**Table 1**). This effect was also observed as Ca^2+^ dissociated from the CaM-peptide complexes by either following the intrinsic Trp fluorescence of the peptides (**Figures 8C,D**) or the change in fluorescence from IAANS-labeled CaMs (**Figures 8E,F**). This data suggests that the C-domains of CaM and SCaM4 bind NADK differently than the C-domain of SCaM1. Although there was minimal influence on the C-domain Ca^2+^ dissociation rate of sCaM4 with NADK, the Ca^2+^ dissociation rates and structural changes observed were similar to those of sCaM1. Thus, the differences in Ca^2+^ binding observed in the absence of the peptide are minimized in the presence of a CaM-binding peptide, and cannot explain the differential regulation of the enzyme. Therefore, we expect that the soybean CaMs should respond similarly to a Ca^2+^ transient in the presence of Mg^2+^ and NADK.

### Exposure of the Plant CaMs to Ca^2+^ Transients in the Presence of Mg^2+^ and NADK

**FIGURE 9:**
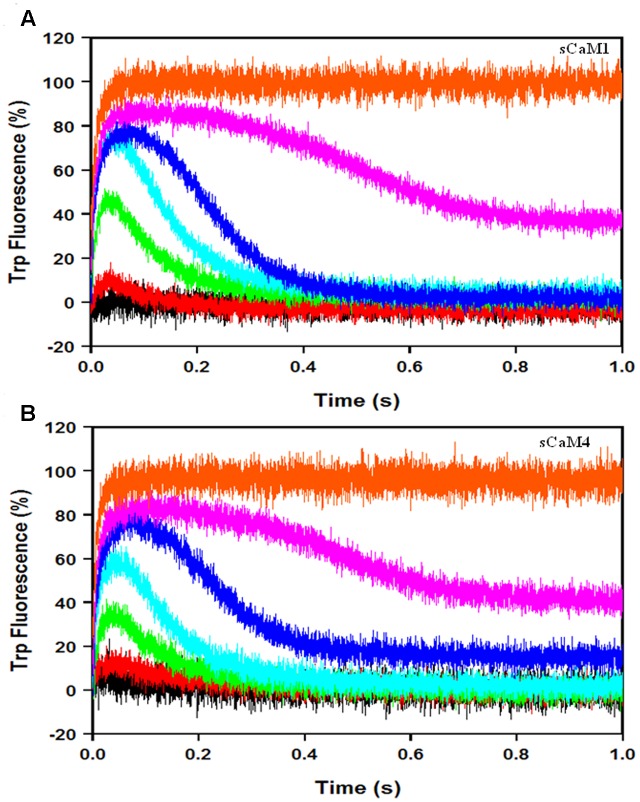
**sCaM1 and sCaM4 response to slow Ca^2+^ transients. (A)** Time course of change in intrinsic Trp fluorescence in the NADK peptide as sCaM1 is transiently occupied by Ca^2+^ in the presence of Mg^2+^. 3 μM WT sCaM1 with 9 μM NADK peptide, 1 mM Mg^2+^ and 500 μM EDTA in stopped-flow buffer (10 mM MOPS, 150 mM KCl, pH 7.0) plus 1mM Mg^2+^ was rapidly mixed with an equal volume of increasing Ca^2+^ [0 μM (black), 10 μM (red), 30 μM (green), 60 μM (cyan), 120 μM (blue), 250 μM (magenta), 2 mM (orange)] in stopped-flow buffer plus 1 mM Mg^2+^ at 20°C. **(B)** Time course of change in intrinsic Trp fluorescence in NADK as sCaM4 is transiently occupied by Ca^2+^ in the presence of Mg^2+^. 3 μM WT sCaM4 with 9 μM NADK, 1 mM Mg^2+^ and 500 μM EDTA in stopped-flow buffer plus 1 mM Mg^2+^ was rapidly mixed with an equal volume of increasing Ca^2+^ (0, 10, 30, 60, 120, and 250 μM, 2 mM) in stopped-flow buffer plus 1 mM Mg^2+^ at 20°C. There was no significant difference between sCaM1 and sCaM4 occupancy under these conditions. The visible occupancy for all traces was determined as described in section “Materials and Methods.”

**Figures 9A,B** show the response of the plant CaMs to artificial Ca^2+^ transients (up to 70 ms half-life) in the presence of physiological Mg^2+^ and NADK peptide following the change in intrinsic Trp fluorescence from NADK. As we predicted, there was no significant difference in the response of either plant CaM to the Ca^2+^ transients (**Table 2**). These data suggest that the plant CaMs may have evolved their differential enzyme regulation without changing their ability to respond to Ca^2+^ signals.

## Discussion

Families of CaM genes that encode unique proteins have been found in numerous plant species including *A. thaliana*, rice, tobacco, and soybean (McCormack and Braam, 2003; Zhao et al., 2013). In the soybean, the sCaM1 and sCaM4 isoforms can differentially activate or inhibit CaM-regulated enzymes from both plants and vertebrates (Lee et al., 1995, 2000). The mechanism by which the various plant CaMs can activate or inhibit select targets is not well understood. There are at least three ways one can envision differential target regulation by CaM: (1) selective target binding (if it doesn’t bind it won’t activate); (2) altered cation binding (either Ca^2+^ and/or Mg^2+^); and (3) perturbation of the bound CaM structure (Van Lierop et al., 2002; Karita et al., 2004).

Using a large CaM protein target microarray for the *Arabidopsis* proteome, the bona fide *Arabidopsis* CaMs were all shown to cluster into a single hub of protein targets (Popescu et al., 2007). Furthermore, both sCaM1 and sCaM4 bind equally well to the reciprocally regulated enzymes MLCK and neuronal nitric oxide synthase (nNOS) (Cho et al., 1998; Kondo et al., 1999; Van Lierop et al., 2002), suggesting the different CaMs can become competitive antagonists of one another. Although there may be a small subset of CaM protein targets that are unique to a particular plant CaM or have drastically different affinities, the vast majority of targets appear to bind the bona fide plant CaMs. Thus, it would appear that the plant CaMs do not necessarily discriminate in their ability to bind targets to reciprocally activate targets.

Based on the differences in Ca^2+^ and Mg^2+^ binding properties of the isolated soybean CaMs (confirmed in this work), it has been proposed that sCaM1 and sCaM4 are tuned to respond to unique Ca^2+^ signals in order to differentially regulate targets (Gifford et al., 2013). However, our results show that due to their nearly identical Mg^2+^ dissociation rates, both proteins responded similarly to rapid Ca^2+^ transients since Ca^2+^ cannot bind until Mg^2+^ dissociates. Furthermore, in cells there is little free CaM in the cytoplasm since CaM is mostly pre-bound to its targets (Maier et al., 2006; Yang et al., 2014). In the presence of both physiological Mg^2+^ and a target peptide, sCaM1 and sCaM4 responded nearly identically to slow Ca^2+^ transients. Thus, for NADK, the only plant enzyme known to be differentially regulated by the sCaMs, the differential regulation does not appear to be due to an altered response to the Ca^2+^ signal.

Our current data with the NADK peptide, as well as previous work, suggest the competitive antagonism of sCaM1 and sCaM4 occurs through an altered mode of CaM binding to the enzymes (Van Lierop et al., 2002; Karita et al., 2004). In the case of MLCK, there does not appear to be a difference in how the CaMs bind the target peptide, but in the subsequent structural change required to activate the enzyme. In fact, the residues responsible for the inhibition of MLCK by sCaM4 are found on the outside surface of sCaM4 rather than in the peptide binding interface (Van Lierop et al., 2002). However, our data suggest that sCaM4 binds the NADK peptide differently from sCaM1, at least at the C-terminal domain. This altered structure may prevent the structural change in the enzyme required for activation.

In summary, although the sCaM1 and sCaM4 isoforms had unique cation binding properties in isolation, in the more physiological situation with competition from Mg^2+^ and the presence of a target peptide, the plant CaMs responded indistinguishably to Ca^2+^ transients. This suggests that the plant CaMs have conserved their response to Ca^2+^ signals throughout their evolution. Thus, the CaMs respond to the same Ca^2+^ signals while only activating certain enzymes through altered modes of binding. This would allow the plant during times of stress to maintain essential CaM activities, activate additional signaling nodes that were not necessary under normal circumstances, and turn off subsets of signaling nodes that might exacerbate the stress. By comparing how naturally occurring CaM isoforms from other species diverge from vertebrate CaM, we can learn more about how changes in the CaM sequence alter downstream target function and cellular responses. In the future, we may be able to smartly engineer CaMs with specific properties that could be used as therapeutics for human disease (Davis et al., 2016; Walton et al., 2016) or better equip plants against biotic or abiotic stressors (Zeng et al., 2015).

## Author Contributions

JD and SW designed the experiments and along with ST wrote the manuscript. SW, HC, and VS performed the stopped-flow experiments. HC, AO, and JS performed the steady-state experiments. BJ developed the CaM^T5C^ constructs.

## Conflict of Interest Statement

The authors declare that the research was conducted in the absence of any commercial or financial relationships that could be construed as a potential conflict of interest.
